# Experimental Optimization of a Plasmonic Surface Biofunctionalization, toward the Bimodal Biosensing and Kinetic Characterization of sPD1

**DOI:** 10.1021/acsomega.5c08871

**Published:** 2025-12-23

**Authors:** Fahd Khalid-Salako, Hasan Kurt, Meral Yüce

**Affiliations:** † Sabanci University Nanotechnology Research and Application Centre, 34956 Istanbul, Türkiye; ‡ Sabanci University Faculty of Engineering and Natural Sciences, 34956 Istanbul, Türkiye; § Department of Bioengineering, Royal School of Mines, 4615 Imperial College London, London SW7 2AZ, U.K.; ∥ Department of Biomedical Engineering, School of Engineering and Natural Sciences, Istanbul Medipol University, Istanbul 34810, Türkiye; ⊥ Research Institute for Health Sciences and Technologies (SABITA), Istanbul Medipol University, Istanbul 34810, Türkiye

## Abstract

Soluble PD1 (sPD1) plays a complex role in cancer pathophysiology, reportedly dependent on its interactions with immune checkpoint proteins and therapeutic monoclonal antibodies. Yet, no biosensor platform currently affords simultaneous quantification and kinetic profiling of sPD1–antibody interactions. Here, we introduce a surface plasmon resonance (SPR) refractometric biosensor setup functionalized with nivolumab that integrates direct, label-free quantification and real-time functional analysis of sPD1 in a buffer and human serum. Sensor functionalization strategies and robust regeneration protocols were investigated and optimized. The biosensor achieved a limit of detection of 5 ng/mL (limit of quantification (LOQ) 8.7 ng/mL; dynamic range 8.7 ng/mL to 376 μg/mL) and quantified sPD1 with 93 ± 5% recovery in 1% serum and 62 ± 30% in 10% serum. Kinetic constants (*k*_a_ ≈ 2.32 × 10^5^ M^–1^ s^–1^; *k*_d_ ≈ 1.03 × 10^–3^ s^–1^; *K*_D_ ≈ 4.66 nM) match literature values for the nivolumab–PD1 interaction. This dual-mode SPR platform represents the first attempt to achieve two distinct analytical functions: (i) quantitative detection of soluble PD-1 (sPD1) and (ii) kinetic characterization of the sPD1–antibody interaction, in a single platform, within biological media. The emergent significance of sPD1 as a liquid biopsy biomarker in immuno-oncologic profiling positions this biosensor setup as a powerful tool for research and potential clinical monitoring of immune checkpoint dynamics.

## Graphical abstract

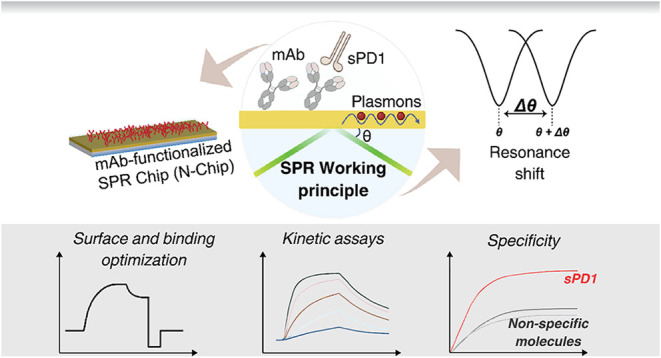

## 1 Introduction

Programmed death-1 (PD1) is a key immune-inhibitory receptor expressed on the surface of activated T cells, B cells, macrophages, and dendritic cells. Through engagement with its ligands PD-L1 and PD-L2, PD1 serves as a critical checkpoint that maintains self-tolerance and prevents autoimmune pathology.^1^ However, many tumors exploit this axis by upregulating PD-L1/PD-L2 to evade immune surveillance and establish an immunosuppressive microenvironment.^2–4^ Soluble PD1 (sPD1), a splice variant lacking the transmembrane domain,^5,6^ circulates as a monomer in the bloodstream and can modulate immune signaling by competing with membrane-bound PD1 for ligand binding. Its precise biological role remains elusive; some studies report that sPD1 enhances antitumor immunity by blocking membrane-bound PD1/PD-L1 and PD-L2 interactions,^6,7^ while others implicate it in T-cell inhibition.^8^ Clinical data consistently link elevated sPD1 levels to disease progression and prognosis across multiple malignancies, autoimmune disorders, and infections.^5,9,10^

Given its dynamic role and prognostic value, the reliable measurement of sPD1 in patient samples has become increasingly important. Traditional approaches, including enzyme-linked immunosorbent assays (ELISA), surface-enhanced Raman spectroscopy (SERS), fluorescent sandwich assays, and electrochemical sensors, have achieved impressive limits of detection (often in the pg/mL range) but provide only end-point concentration readouts.^11–14^ They do not reveal the kinetic parameters that govern antibody–antigen interactions and are important for in-depth characterization of the sPD1–therapeutic anti-PD1 antibody binding. Post-translational modifications of circulating sPD1, potential competition with other ligands, and individual patient variation can all influence the functional binding behavior of sPD1;^15^ information is lost in concentration-only assays, which may strengthen mechanistic understanding for clinical monitoring.

Surface plasmon resonance (SPR) refractometric biosensors overcome this limitation by providing label-free, real-time monitoring of biomolecular interactions.^16^ Changes in the refractive index near the gold-coated surface, directly proportional to mass changes from binding events, are recorded as resonance shift-derived response units (RU) that trace association and dissociation phases of the response-time plot. SPR has previously been used to characterize PD1/PD-L1 interactions in drug-screening contexts^17,18^ and to develop biosensors for soluble proteins such as VEGF and TNF-α,^19,20^ yet there remains no report of a biosensor platform that concurrently quantifies sPD1 and profiles its binding kinetics with a therapeutic antibody.

Moreover, the immobilization strategy profoundly affects assay performance. Ligand orientation, surface density, steric accessibility, and regeneration stability all hinge on the chemistry used to affix the capture molecule. For IgG1-type antibodies, site-specific capture via Fcγ receptors or protein G has been employed to orient the molecule, maintaining optimal antigen-binding fragment (Fab) accessibility.^19–21^ However, IgG4-type antibodies like nivolumab exhibit 4- to 40-fold lower affinity for FcγRI compared to IgG1 and are engineered (through S228P mutation) to minimize FcγR engagement, complicating FcγRI-mediated capture.^22,23^ Alternatively, conventional amine coupling, where surface carboxyl groups are activated with NHS/EDC to form covalent bonds with antibody amines, offers robust immobilization but may randomize orientation, impair antigen-binding accessibility, and introduce steric hindrance.

In this work, we address these gaps by investigating and optimizing an immobilization strategy for nivolumab as the biorecognition component of the sPD1 biosensor. Furthermore, we leverage the real-time signal transduction modality of SPR refractometric biosensors to comprehensively characterize the kinetics of binding events between surface-immobilized nivolumab (OPD) and sPD1. We also evaluate the detection and quantification capacity of sPD1 analyte by the OPD-functionalized sensor in buffer and serum matrix solutions to establish clinical validity.

## 2 Results and Discussion

The biosensor surface was modified with nivolumab (OPD) as an affinity probe of sPD1. Two functionalization approaches were assessed:

- Capturing OPD on an FcγRI-immobilized surface, leveraging interactions between the antibody’s Fc region and FcγRI to achieve site-oriented capture (F-chip).
- Directly immobilizing the OPD molecules by covalent amine coupling (N-chip).

### 2.1 Sensor Surface Preparation and OPD-Functionalization

The F-chip and N-chip were prepared following immobilization chemistries rooted in established principles. The F-chip was prepared based on the FcγRI-mediated capture of OPD, reproducing similar setups developed in our group.^19,20^ The F-chip was prepared using a sensor chip in which the dextran matrix has been precoated with streptavidin (SA). Prebiotinylated FcγRI ectodomain fragments are immobilized on the chip, leveraging the strong affinity of streptavidin for biotin. Subsequently, the monoclonal antibody (mAb)–OPD is captured on this surface, leveraging the binding of FcγRI to the OPD-Fc region. Conversely, the N-chip is prepared using a simple amine coupling chemistry to covalently bind the OPD to activated carboxymethyl groups on an uncoated sensor chip dextran matrix (CM5).

Importantly, the surface ligand density was optimized: FcγRI was captured on the F-chip at targeted levels of 150 RU, following experimental optimizations done for steric hindrance minimization, while the OPD was consistently captured by 120 s injections of a 90 nM solution of the mAb in fresh running buffer. On the other hand, OPD was directly immobilized on the N-chip at a targeted level of 300 RU for kinetic analyses and 1500 RU for sensitivity and selectivity studies, accounting for the random immobilization orientation of the molecule, the low possibility of steric hindrance, given that sPD1 is a much smaller protein than OPD, and the consequent sensitivity challenge, arising from SPR being a mass-sensitive technique. Surface preparation sensorgrams, including FcγRI immobilization and OPD capture single-cycle kinetics of the F-chip, and direct OPD immobilization on N-chip are presented in Figure 1.

**Figure 1:**
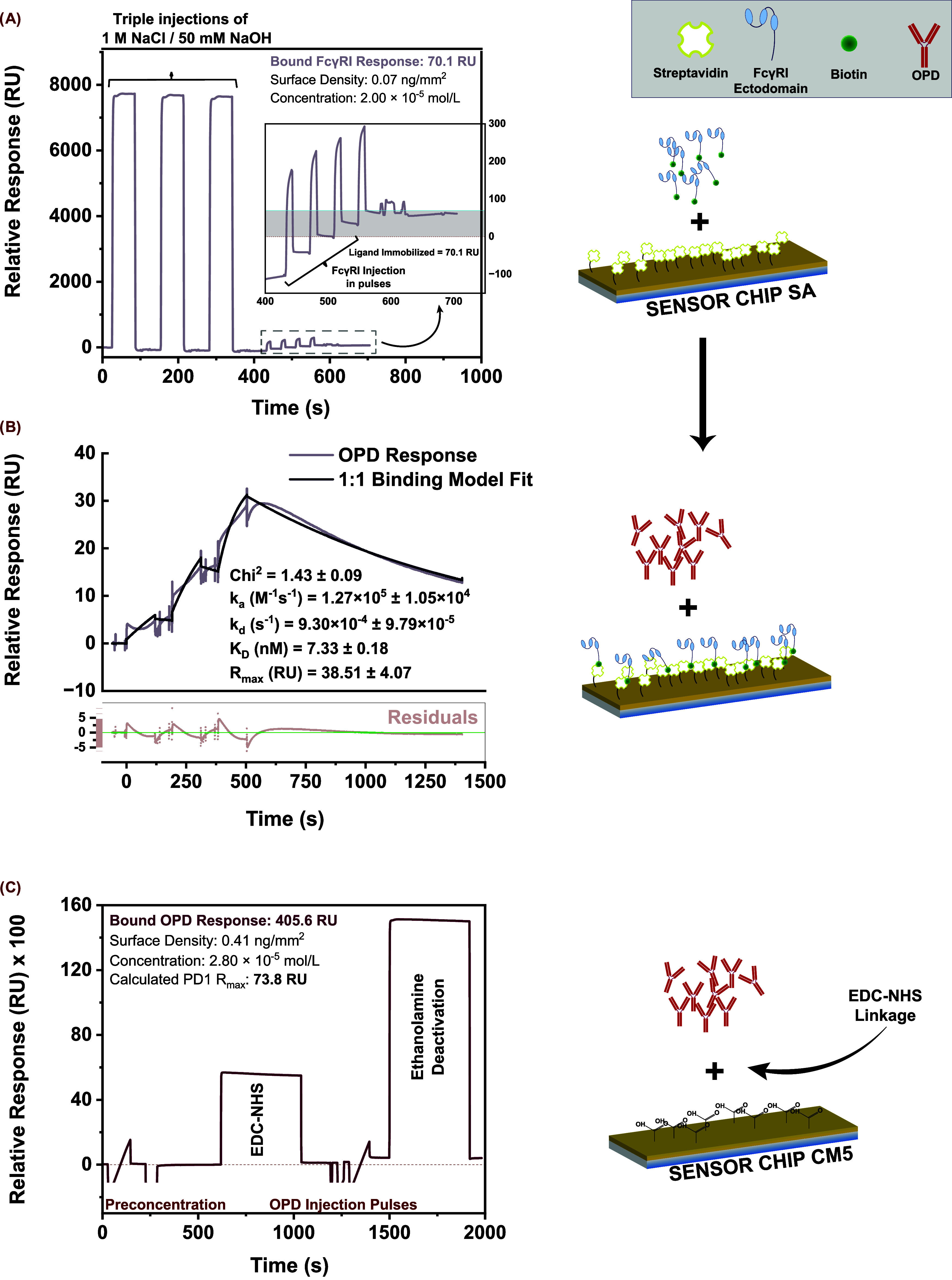
Sensor surface preparation sensorgrams. (A) Triple injections of NaCl/NaOH (1 M NaCl + 50 mM NaOH, 60 s each) over a streptavidin-coated SA chip (baseline ∼0 RU), followed by five FcγRI injections (biotinylated CD64; target immobilization ≈ 150 RU) in pulses (inset). Inset shows an enlarged region (400–750 s) of the FcγRI capture run, with the fitted immobilization level (70.1 RU), calculated surface density (0.07 ng/mm^2^), and corresponding molar surface concentration (2.0 × 10^–5^ mol/L). (B) Single-cycle kinetics of nivolumab (OPD) capture on FcγRI-immobilized surface: three sequential OPD injections (90, 30, 10 nM; 120 s contact, 900 s dissociation) fitted to a 1:1 Langmuir model. Reported fit parameters (mean ± SD, *n* = 3) include χ^2^ = 1.43 ± 0.09, *k*_a_ = 1.27 × 10^5^ ± 1.05 × 10^4^ M^–1^s^–1^, *k*_d_ = 9.3 × 10^–4^ ± 9.8 × 10^–5^s^–1^. *K*_D_ = 7.33 ± 0.18 nM, and *R*_max_ = 38.5 ± 4.1 RU. Residuals are shown beneath the main trace. (C) Amine coupling chemistry on a CM5 chip for direct OPD immobilization (N-chip): (i) preconcentration at pH 4.5, (ii) EDC/NHS activation of carboxymethyl dextran (∼500–600 RU), (iii) three OPD injection pulses (50 nM) reaching a bound response of 405.6 RU (surface density = 0.41 ng/mm^2^; concentration = 2.8 × 10^–5^ mol/L; calculated *R*_max_ for sPD1 = 73.8 RU), and (iv) ethanolamine deactivation. Time axis origin set at the start of conditioning.

Schematics on the right panel illustrate the surface preparation workflows corresponding to the biotinylated FcγRI immobilization and OPD capture in A and B (F-chip) and the direct, random immobilization of the OPD by EDC-NHS coupling in C (N-chip).

Temporal regions of the sensorgrams corresponding to important steps of the respective processes are indicated, including the triple injections of 1 M NaCl/50 mM NaOH solution, injection of FcγRI in pulses (Figure 1A), and a single-cycle kinetics assay demonstrating the FcγRI capture of OPD (Figure 1B) in the F-chip sensor surface preparation. The single-cycle kinetics assay sensorgram was fitted to a 1:1 Langmuir binding model using the Biacore Evaluation software (3.0 Biacore T200, Shrewsbury, MA). The sensorgram illustrated in Figure 1B is obtained as a time-series plot of average RU from triplicate cycles fitted separately and averaged. Parameters and kinetic constants are presented as the mean ± SD (*n* = 3). The kinetic affinity value reported in our study corresponds to earlier SPR and biolayer interferometry studies reporting the affinity of IgG4-type antibodies to FcγRI in the nanomolar range.^23,24^ Importantly, the shape and curvature of the sensorgram, as well as rate constants obtained, signify successful OPD capture at 90 nM under the adopted injection conditions (120 s; 30 μL·min^–1^).

Similarly, important regions of the sensorgram in the N-chip preparation procedure (Figure 1C) are indicated, including preconcentration, EDC/NHS activation of the dextran COO^–^ groups, OPD pulsatile injection, and ethanolamine deactivation. Having set a 300 RU immobilization target, the final immobilized ligand level obtained after three pulses was 405.6 RU. Assuming a 1 RU response corresponds to 1 pg/mm^2^ surface density and a 100 nm dextran matrix thickness as specified by the instrument and chip manufacturer.^25^ The final immobilization response corresponds to a surface density of approximately 0.41 ng/mm^2^ and a concentration of 2.84 μM. Theoretically, we calculate a maximum response (*R*_max_) for sPD1 of 74 RU. The *R*_max_ value indicates the maximum response achievable on injection of sPD1 at infinite concentration and over an infinite contact time, assuming a 1:1 stoichiometry, in principle. We expect to reach lower response values for sPD1 due to the nonspecific orientation of the immobilized OPD molecules (the abundance of amine groups in the mAb randomizes the orientation of the molecule upon covalent attachment to the sensor surface) and other experimental conditions, including the OPD ligand activity and sPD1 analyte injection parameters.

### 2.2 Regeneration Studies

#### 2.2.1 F-Chip Regeneration

FcγRI immobilization on the F-chip by streptavidin–biotin capture is considered permanent immobilization, given that the high affinity of streptavidin for biotin results in a bond nearly as strong as a covalent bond. Therefore, the strategy we adopted to regenerate the F-chip surface was to detach the captured OPD from FcγRI, similarly to Khaligh et al.^20^ There is a dearth of information on suitable regeneration conditions for a biotinylated FcγRI-immobilized surface following OPD capture. This is due, partly, to the novelty of the setup and prevalence, instead, of OPD capture in literature, by alternative methods such as antihuman Fc mAb and protein G.^26–29^ Crucially, OPD, being an IgG4 mAb, differs from some mAbs used previously as affinity probe components of SPR biosensors, such as bevacizumab^20^ and adalimumab,^19^ both IgG1–type, in their respective Fc region amino acid sequences and FcγRI binding peculiarities.^23^ It is therefore imperative to experimentally establish an optimal regeneration condition that suitably removes bound OPD, while preserving FcγRI activity. For this purpose, six regeneration conditions were scouted preliminarily, including 10 mM glycine–HCl buffer (pH 3), 10 mM acetate buffers (pH 4.5, 5.0, and 5.5), 5 M NaCl solution, and a 100% ethylene glycol solution. Additionally, we scouted blank running buffer–HEPES-buffered saline 1× with EDTA and surfactant p20 (HBS-EP 1× pH 7.4) as a seventh regeneration condition, testing a hypothesis of spontaneous OPD dissociation, based on the low-affinity binding of the mAb to FcγRI and fast dissociation, which has been reported in the literature,^23^ and observed during the F-chip surface preparation (Figure 1B). We present trends in baseline responses and responses at equilibrium (*R*_eq_) of each regeneration condition over five consecutive cycles in Figure 2. We also calculated coefficients of variation (CV%) for the observed trends with the equation: CV% = (x̅/σ) × 100% [where σ = the standard deviation; x̅ = the average].

**Figure 2:**
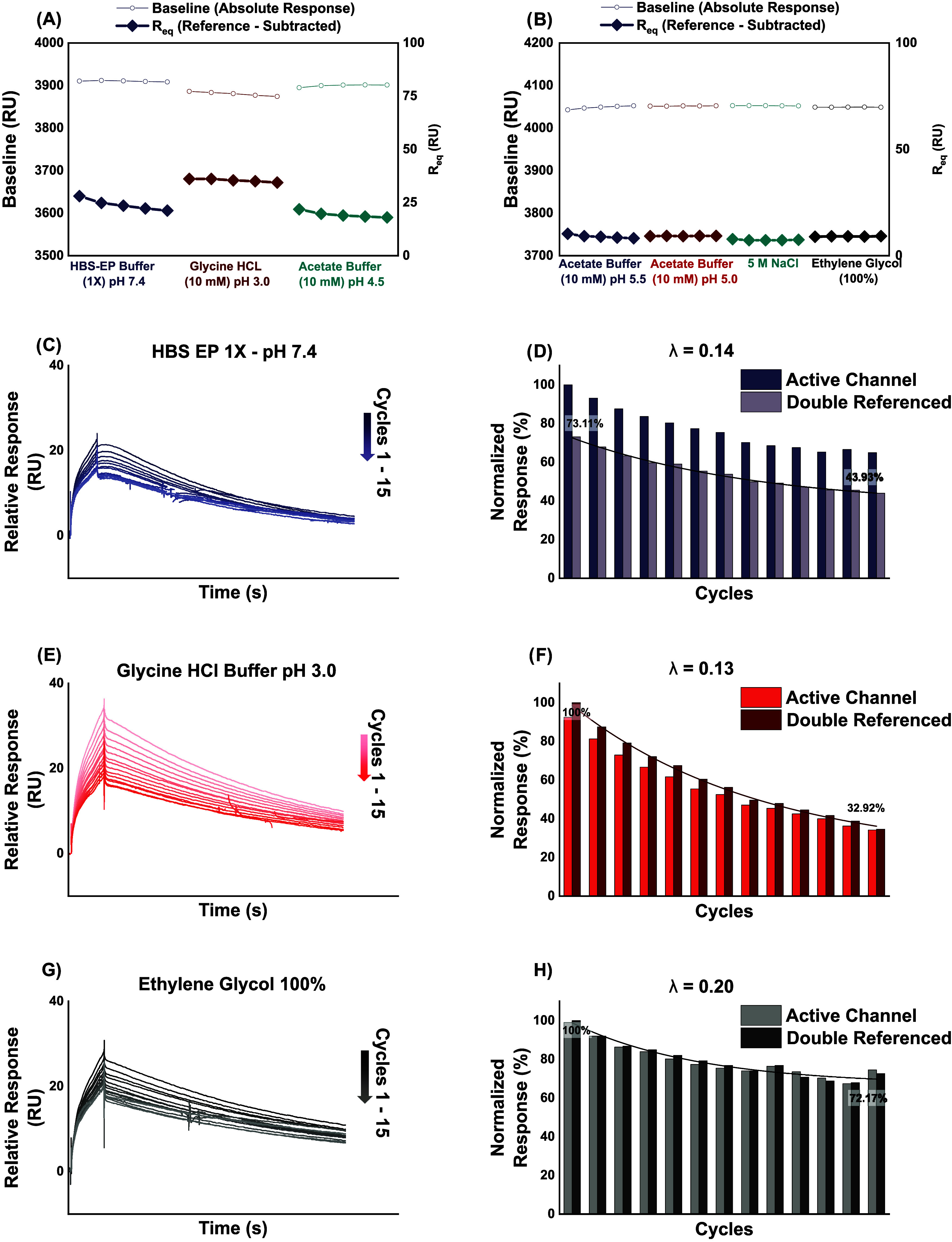
Regeneration scouting and verification on the F-chip. Scouting phase (five cycles): Baseline (○, left *y*-axis) and equilibrium binding response *R*_eq_ (◆, right *y*-axis; reference-subtracted) trends for seven candidate regeneration conditions. (A) HBS-EP 1× running buffer (pH 7.4), 10 mM glycine–HCl (pH 3.0), and 10 mM acetate (pH 4.5). (B) 10 mM Acetate (pH 5.0 and pH 5.5), 5 M NaCl, and 100% ethylene glycol. Each cycle comprised injection of 90 nM OPD (120 s at 30 μL/min), 30 s regeneration, and 60 s stabilization. Verification phase (15 cycles): Sensorgrams (left) and normalized bar plots (right) for three selected conditions. Double-referenced bar heights (darker fill) are overlaid on active-channel responses (lighter fill), and exponential decay fits (solid lines) report decay constants λ. (C, D) (HBS-EP 1×): Spontaneous regeneration (1800 s dissociation). λ = 0.14; double-referenced *R*_eq_ declines from 73.1 to 43.9%. (E, F) (glycine–HCl pH 3.0 + 2.5 M NaCl): Chemical regeneration (30 s at 30 μL/min). λ = 0.13; double-referenced *R*_eq_ declines from 100 to 32.9%. (G, H) (100% ethylene glycol): Chemical regeneration (30 s at 30 μL/min). λ = 0.20; double-referenced *R*_eq_ declines from 100 to 72.2%.

All traces are baseline-adjusted and double-referenced by subtracting the reference channel and blank injections. Time axes are aligned with the start of each OPD injection.

From the findings of the regeneration scouting studies, the blank running buffer condition (HBS-EP 1×) notably led to a progressive decrease in *R*_eq_ (CV% = 11.04%), which was seemingly restored with the glycine pH 3 solution (CV% = 2.7%), accompanied by a concurrent decrease in baseline. Ten millimolars of acetate buffer at pH 5.5, 5.0, and 4.5 all resulted in a progressive decrease in binding activity, with concurrent baseline increase to varying degrees. Similarly, the 5 M NaCl solution resulted in a decrease in binding activity (*R*_eq_) as well as a baseline (CV% = 3.34 and 0.03%, respectively). On the other hand, ethylene glycol demonstrated restoration of binding activity.

Following the results of the regeneration scouting study, 10 mM glycine buffer (pH 3) and 100% ethylene glycol were advanced to the regeneration verification study. Additionally, a third regeneration verification study was conducted without a regeneration solution, allowing for a dissociation time (blank running buffer injection) of up to 30 min to further explore our spontaneous regeneration hypothesis. Each condition was applied to regenerate the sensor surface for 15 consecutive cycles. *R*_eq_ values obtained from both blank and sample injections over the active and reference flow channels were normalized, with the maximum *R*_eq_ set to 100%. All responses below zero were recorded as 0 RU. Normalized *R*_eq_ values obtained from the active flow channel (Fc2), as well as normalized double-referenced *R*_eq_ values from at least 13 of the 15 cycles, are presented in Figure 2. Baseline-adjusted sensorgrams of the sample injection cycles for each regeneration condition are also presented in Figure 2.

The exponential decay constants obtained with the HBS-EP 1× running buffer over a 1800 s dissociation period (λ = 0.14) are comparable with decay constants obtained with both 10 mM glycine–HCl buffer pH 3 (λ = 0.13) and 100% ethylene glycol solution (λ = 0.2). Additionally, the HBS-EP 1× dissociation resulted in a total 29.18% reduction in surface activity, measured by the normalized double-referenced response variation over the 13 cycles recorded. This is comparable to the decrease recorded with ethylene glycol (27.83%) and much lower than the 67.98% observed with glycine–HCl buffer (pH 3). Sensorgrams obtained from the regeneration verification studies also generally exhibit time-based exponential reductions in response, at the end of the OPD injection phase, implying significant dissociation of the mAb from the FcγRI ectodomain, after capture (Figure 2C,E,G). Visual observation of the sensorgram demonstrates only minimal divergence from cycle 1 to cycle 15 in the case of HBS-EP buffer 1× (Figure 2C) and ethylene glycol (Figure 2G). In contrast, 10 mM glycine–HCl buffer (pH 3) recorded significantly higher sensorgram divergence (Figure 2E)

In general, neither ethylene glycol nor glycine buffer demonstrates significantly better regeneration compared with spontaneous dissociation. These findings affirm the appropriateness of spontaneous dissociation as a sensor surface regeneration strategy in this setup, having demonstrated comparably effective regeneration and minimizing flow system contamination associated with other regeneration conditions, considering the fact that HBS-EP 1× pH 7.4, being the running buffer, contributes no additional refractive index.

#### 2.2.2 N-Chip Regeneration

Our approach to regenerating the N-chip involved detachment of the sPD1 analyte from the covalently immobilized OPD ligand at the end of the cycle. While nivolumab has once, reportedly, been covalently immobilized to comparatively study its PD1 binding kinetics on different biosensor platforms in the literature,^30^ the condition adopted for regenerating the sensor surfaces was not reported in that paper. There are also no established regeneration strategies in the literature for such a setup. Accordingly, we draw on an understanding of the chemistry underlying OPD-PD1 ectodomain binding as a starting point for uncovering regeneration strategies that maximize regeneration with minimal impact on surface binding activity.

Tan et al.^28^ reported the key structural features of the nivolumab–PD1 complex, noting that complex stability depends on all three CDR loops constituting the variable heavy chain of OPD; CDR2 and CDR1 loops of the mAb’s variable light chain; the FG and BC loops of PD1; as well as a novel N-loop of the PD1 protein ectodomain, spanning the L25 to P34 positions. Crucially, the stability of the complex is provided by 16 hydrogen bonds, 10 of which are formed between the PD1 N-loop and the CDR1 and CDR2 of the OPD variable heavy chain, with the BC and FG loops of the protein IgV domain contributing one and five hydrogen bonds, respectively.^28^

It is expected that acidic conditions, pH lower than the isoelectric points of both OPD (6.1–8.5) and PD1 (8.05), would result in electrostatic repulsion due to the net positive charge of both molecules, while high ionic conditions would cause competitive displacement of the hydrogen bonds.^31^ However, the optimal regeneration conditions can only be experimentally determined. We scouted three regeneration conditions, including a sequential injection of 10 mM glycine hydrochloride buffer (pH 3) followed by a 2.5 M NaCl solution (Glycine + NaCl); a similar injection of 10 mM acetate buffer pH 5.0 and 2.5 M NaCl (acetate + NaCl); a single 5 M NaCl solution injection. Regeneration scouting and verification studies were conducted similarly to those of the F-chip. Findings of the N-chip regeneration studies are presented in Figure 3.

**Figure 3:**
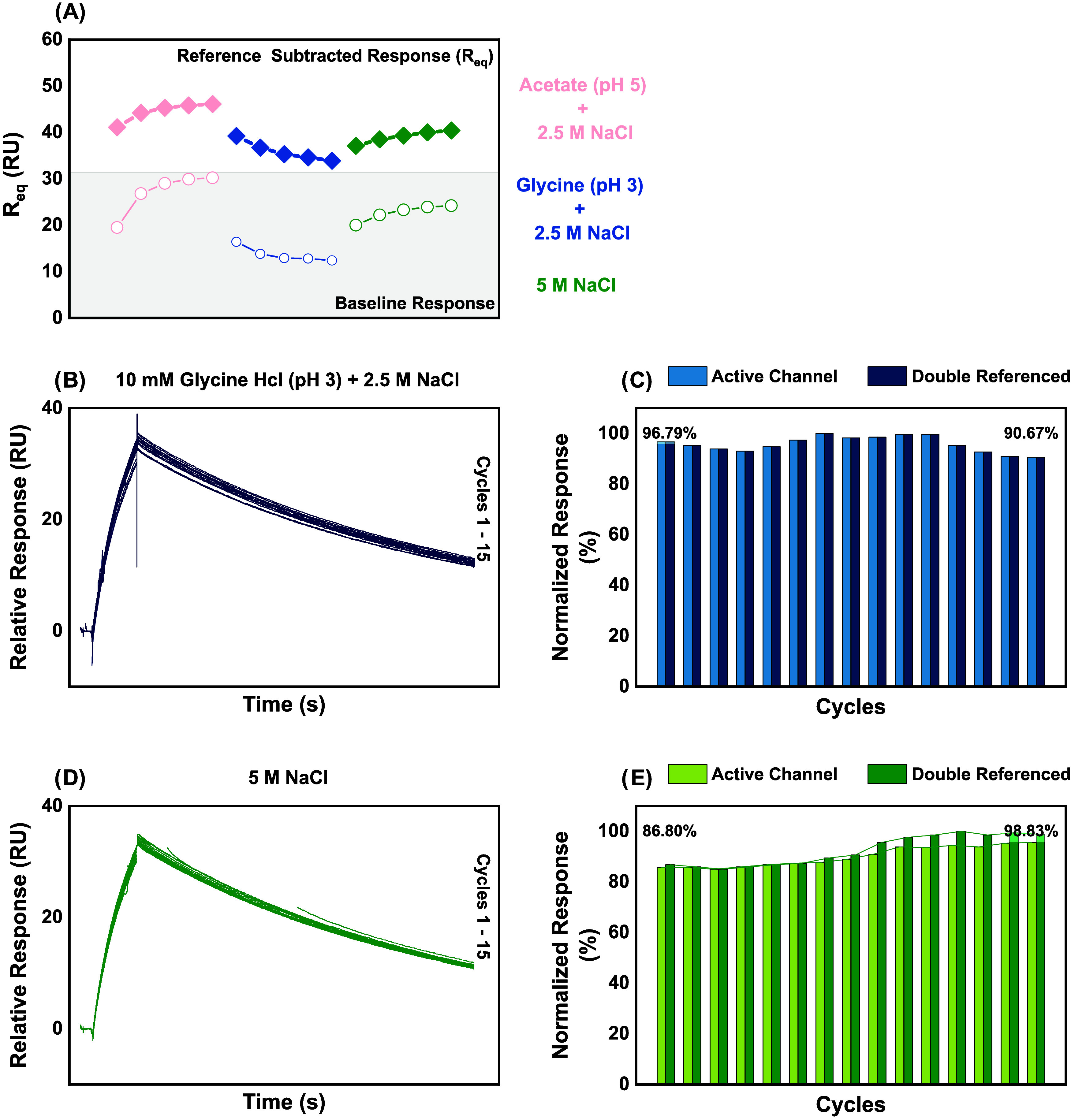
Regeneration performance of the N-chip following sPD1 analyte binding (A) scouting phase: Baseline (open symbols) and equilibrium binding response (*R*_eq_, filled symbols; reference-subtracted) trends over five consecutive cycles of 60 nM sPD1 injection (120 s contact at 30 μL/min), each followed by regeneration (30 s at 30 μL/min) with three candidate conditions: 10 mM glycine–HCl (pH 3.0) + 2.5 M NaCl (teal), 10 mM acetate (pH 5.0) + 2.5 M NaCl (cyan), and 5 M NaCl (maroon). (B) Verification phase with glycine + NaCl (15 analyte injection cycles). Baseline-adjusted sensorgrams for cycles 1–15 showing overlay of sPD1 binding and dissociation. (C) Verification phase with glycine + NaCl (15 analyte injection cycles). Bar plot of normalized responses (active channel, dark teal; double-referenced, light teal). Percent retention of double-referenced *R*_eq_: 96.8% (cycle 1) to 90.7% (cycle 15); coefficient of variation CV% = 5.8%. (D) Verification phase with 5 M NaCl (15 analyte injection cycles). Baseline-adjusted sensorgrams for cycles 1–15. (E) Verification phase with 5 M NaCl (15 analyte injection cycles). Bar plot of normalized responses (active channel, dark maroon; double-referenced, light maroon), showing retention from 86.8% (cycle 1) to 98.8% (cycle 15); CV = 3.4%.

The acetate + NaCl regeneration condition demonstrated progressively increasing baseline (CV% = 16.4%) and equilibrium response (CV% = 4.57%), demonstrating surface accumulation and inefficient regeneration. Conversely, glycine + NaCl and 5 M NaCl appeared to recover and maintain baseline (CV% = 11.82 and 7.89%, respectively) and equilibrium responses (CV% = 5.83 and 3.36%, respectively), demonstrating regeneration efficacy with relatively minimal binding activity disruption. Accordingly, regeneration verification studies were conducted for the glycine + NaCl and 5 M NaCl conditions, respectively. Both regeneration conditions appear to maintain binding activity over the 15 cycles, as demonstrated in minimal sensorgram divergence (Figure 3B,3D), and low variations in the normalized response plots (Figure 3C,3E), where a 6.12% variation in surface activity (96.79–90.67) was observed with glycine + NaCl, while 5 M NaCl produced a 12.02% variation. These findings lend experimental credibility to the hypothesis that low pH and high salt concentration conditions can sufficiently denature the OPD-sPD1 complex with minimal damage to the binding activity of the complex, as proposed. Relying on these findings, we adopted glycine + NaCl as the N-chip regeneration condition for subsequent studies.

### 2.3 sPD1 Detection and Kinetics Assay

We characterize sPD1–OPD binding using single-cycle kinetics SPR assays on both the N-chip and the F-chip. This provides concrete evidence of sPD1 detection by the biosensor setups and offers important quantitative information on the functional properties of sPD1 based on its mAb binding kinetics that puts its clinical significance into perspective. Sensorgrams from both the reference flow cell and blank injection cycles were subtracted from the binding curves obtained in the active flow channel from sPD1 analyte injection to derive double-referenced sensorgrams that were fitted with the 1:1 Langmuir binding model. Kinetic association, dissociation, and affinity constants were obtained as presented in Figure 4.

**Figure 4:**
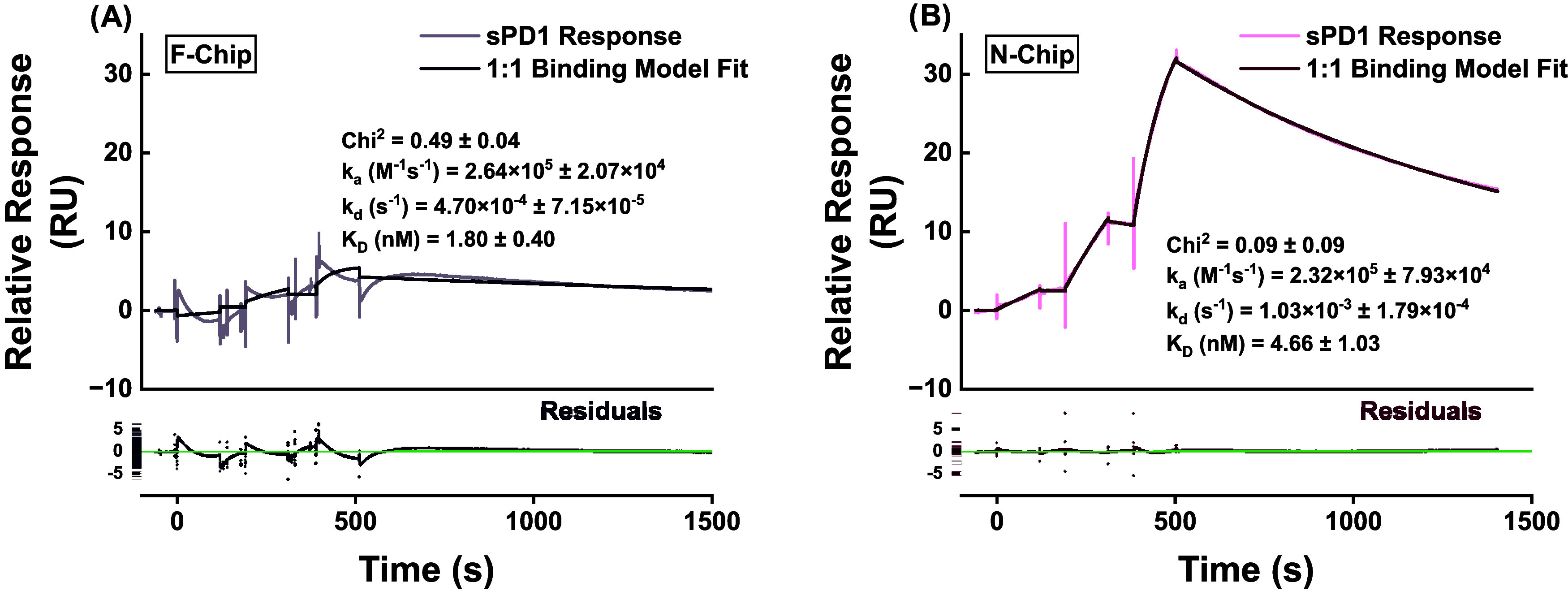
Single-cycle kinetics of sPD1 binding to OPD on F-chip and N-chip. Parameters and kinetic constants are presented as mean ± SD (*n* = 3). (A) F-chip (FcγRI-captured nivolumab): Three sPD1 injections (90, 18, 3.6 nM; 120 s contact at 30 μL/min) with spontaneous regeneration (1800 s dissociation). Double-referenced sensorgrams (subtracting reference channel and blank runs) showing minimal binding response (−9 to 16 RU), consistent with low OPD capture levels (25–30 RU) and rapid OPD dissociation from FcγRI.1:1 Langmuir fit (dark line) yields *k*_a_ = 2.64 × 10^5^ M^–1^s^–1^, *k*_d_ = 4.7 × 10^–3^ s^–1^, and *K*_D_ = 1.8 nM (χ^2^ = 0.5). The poor visual fit and residuals plot (lower panel) call the calculated constants to question and highlight the suboptimal stability for functional assays. (B) N-chip (amine-coupled nivolumab): three sPD1 injections (60, 15, 3.75 nM; 120 s contact at 30 μL/min) with regeneration by 10 mM glycine–HCl (pH 3) + 2.5 M NaCl (30 s). Double-referenced sensorgrams exhibit robust, concentration-dependent binding (0–40 RU) and distinct association and dissociation phases. Global 1:1 Langmuir fit (dark line) yields *k*_a_ = 2.32 × 10^5^ ± 7.93 × 10^4^ M^–1^ s^–1^, *k*_d_ = 1.03 × 10^–3^ ± 1.79 × 10^–4^ s^–1^, *K*_D_ = 4.66 ± 1.03 nM, and *R*_max_ = 41.57 ± 3.26 RU (χ^2^ = 0.09). Residuals (lower panel) confirm excellent fit quality.

On visual inspection, the sPD1 binding recorded on the F-chip appeared to diminish exponentially over the analyte injection period, resulting in concave curvature at the analyte injection periods. This behavior is suspected to arise from the instability of OPD capture by FcγRI, consistent with the kinetic affinity and dissociation constants derived in Figure 1B. Models of nivolumab binding to the PD1 ectodomain reported in the literature^28^ do not indicate binding-induced refolding of the antibody, which would otherwise suggest affinity alterations arising from sPD1 binding, different from those recorded in the regeneration studies. Additionally, the binding curves remained within a relatively small response scale (−9 to 16 RU), appearing indistinguishable from the noise of bulk refractive index change-related spikes at injection start and stop time points. These run contrary to expectations of captured OPD strongly and meaningfully binding sPD1 to generate distinctly measurable binding events.^26–28^ We hypothesize, therefore, that these phenomena arise from OPD-captured surface instability due to the relatively low affinity of OPD-Fc for FcγRI, being an IgG4-type mAb.^23^ OPD capture levels ranged from 29.6 to 25.2 RU on the F-chip, corresponding to a calculated sPD1 *R*_max_ (5.5–4.7 RU). The low capture levels observed, combined with suboptimal capture stability and rapid spontaneous dissociation, impair the sustenance and strength of the sPD1 binding event, undermining the suitability of the F-chip setup for reliable sPD1 detection.

On the other hand, double-referenced sensorgrams obtained with the N-chip demonstrated sustained and distinctly measurable binding events over the sPD1 analyte injection period, followed by a relatively steady dissociation period. We optimized the analyte concentrations and injection parameters for the N-chip, prioritizing kinetic evaluations. This resulted in consistent *k*_a_, *k*_d_, and *K*_D_ values over three repeat cycles. The 1:1 binding model demonstrated a good fit for the sensorgram obtained (χ^2^ = 0.09), and the kinetic constants obtained are similar to those reported earlier in the literature, including the nivolumab EMA assessment report,^32^ and other immunological studies, reporting the OPD binding affinity to PD1 protein in the nanomolar range (1.45–3.6 nM), with *k*_a_ and *k*_d_ values in orders of 10^5^ and 10^–4^, respectively.^26–28^ Mass transport limitation, an artifact of kinetic assays resulting from diffusion rate-limited binding in systems where binding rate exceeds flow rate, was computed in the kinetic assays with a mass transport coefficient *t*_c_, which was obtained in the order of 10^8^ in the kinetic assays.^20^ This indicates the low contribution of diffusion-limited binding to the *k*_a_ values obtained.^33^ Importantly, we also quantified the functional efficiency of the N-chip as the ratio of experimental to theoretical/calculated *R*_max_. Experimental *R*_max_ values from the triplicate assays ranged from 39.68 to 45.33 RU, corresponding to a functional efficiency of 53.62–61.26% (theoretical *R*_max_ – 74 RU). The realized efficiency illustrates high functionality, considering the random orientation of the OPD molecules on the surface and experimental limitations on flow rate and contact time, which all affect the experimental *R*_max_.

Unlike the F-chip, the N-chip recapitulates in vivo binding activity between OPD and sPD1, reproducing binding kinetic constants similar to those earlier reported in *in vitro* studies for membrane-bound PD1. For this reason, the N-chip was adopted for subsequent experiments, while the F-chip was discarded. Nonetheless, we have included the results of experiments conducted on the F-chip up to this point for transparency and to further bolster understanding of the biosensor surface functionalization optimization, by showing what has not worked and potentially informing further studies that may seek to rectify the pitfalls we encountered.

### 2.4 sPD1 Quantification Studies

We evaluated the sensitivity of the OPD-functionalized SPR sensor at serialized concentrations that cut across physiological and typical pathological serum concentrations of sPD1 reported in the literature.^34^ sPD1 solutions ranging from 15 pg/mL to 1.5 μg/mL were injected over the sensor surface at optimized injection parameters (10 μL/min flow rate; 180 s contact time) in triplicate. A calibration curve of plasmonic response (*R*_eq_) against concentration was obtained by fitting the responses with a four-parameter logistic function, using Origin 2025 software (OriginLab Corporation, Northampton, MA). Having established the dynamic range of the biosensor, we further conducted linearity analysis. Control samples with concentrations within the dynamic range were injected over the sensor surface at the same injection parameters, and the calculated concentrations corresponding to the responses obtained were computed, using the equation of the calibration curve. The calibration curve and linearity analysis findings are illustrated in Figure 5.

**Figure 5:**
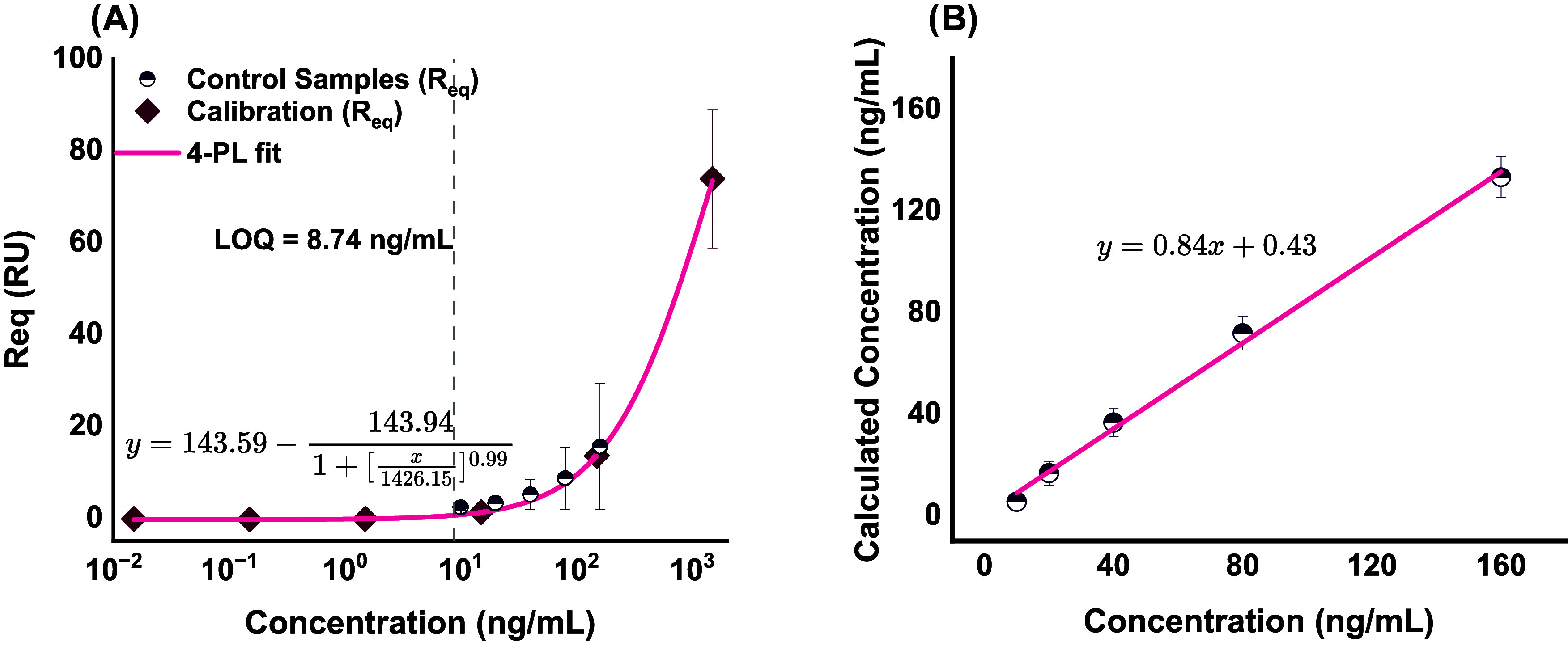
Sensitivity and linearity analyses of the N-chip for sPD1 quantification: (A) calibration curve showing equilibrium SPR response (*R*_eq_) versus sPD1 concentration (0.015–1500 ng/mL), fitted with a four-parameter logistic model (magenta line). The lower limit of quantification (LOQ) at 8.74 ng/mL is indicated by the dashed vertical line. Calibration points (◆) and control samples (●) include ± SD error bars (*n* = 3). (B) Linearity within the dynamic range: calculated concentration (*y*-axis) versus nominal concentration (*x*-axis) for control samples. Linear regression (black line) demonstrates strong agreement (*R* ^2^ = 0.995). Data points (○) include ± SD (*n* = 3).

Limits of blank, detection, and quantification for the biosensor were calculated using the formula:^35^ *y*_0_ + (*c* × σ_0_), where *y*_0_ is the lower asymptote of the calibration curve, σ_0_ is the standard deviation of responses obtained from the 0 ng/mL (blank buffer) solution, following triplicate injections, and *c* is a confidence term that implements the statistical significance of the response relative to background noise. The confidence term (*c*) is often set to 1.65 in the limit of blank calculations and 3.33 and 10 for the limit of detection (LOD) and LOQ, respectively.^20^ The LOD and LOQ values have translational significance in their quantification of the lowest analyte concentration that can be reliably detected and quantified, respectively.

The antibody-functionalized biosensor demonstrated an LOD of 5 ng/mL (∼0.19 nM) and an LOQ of 8.74 ng/mL (∼0.33 nM). Notably, only a few sPD1 biosensors have been reported in the literature, including electrohydrodynamic, electrochemical, fluorescent, surface-enhanced Raman spectroscopy (SERS), and enzyme-linked immunosorbent assay (ELISA) platforms.^11–14^ The LOD values reported with these biosensors are typically in the picogram per milliliter range, which is expectedly lower than the LOD obtained in this study. SPR signal transduction relies on mass-driven refractometric plasmonic resonance shifts, which, given the low relative molecular weight of sPD1 and one-to-one binding stoichiometry, introduces a constraint on detection at lower concentrations. It is also noteworthy that these other biosensors reported in the literature incorporate signal amplification through electrochemistry^13^ or sandwich assays with fluorescent,^12^ colorimetric,^14^ or nanoplasmonic labels,^11^ in contrast with the label-free, direct binding modality of the setup adopted for this study. A comparison of the current study to similar PD1 biosensors in the literature is illustrated in Table 1.

**Table 1:** Key Features of sPD1 Biosensing Platforms Reported in Literature

biosensing technique	detection limits	advantages	limitations	refs
sandwich surface-enhanced Raman spectroscopy	LOD: 6.17 pg/mL	a highly sensitive platform with multiplexing capacity, capable of detecting soluble immune checkpoint biomarkers at subclinical concentrations.	multiple, complex sample preparation steps. The platform also offers no functional binding characterization.	^11^
antibody-based testing on a probe biosensor with fluorometric read-out	dynamic range: 2–3000 pg/mL	highly sensitive with a wide dynamic range and short assay time	the setup employs static functionalization and readout steps, making continuous monitoring tedious and nearly impossible	^12^
a dual-channel electrochemical biosensor	LOD: 10 pg/mL	highly sensitive with a wide linear range. An electrochemical detection module enables data transmission to a smartphone for point-of-care applications.	the biosensor provides end-point quantification like other biosensors, without the necessary binding activity characterization	^13^
	dynamic range: 50–50,000 pg/mL	low-cost materials were used in the biosensor fabrication		
nanofluidic electrohydrodynamic biosensor with colorimetric read-out	LOD: 5 pg/mL	electrohydrodynamic nanofluidic mixing minimizes nonspecific binding. The biosensor is also functionalized to simultaneously detect other immune checkpoint proteins	fluid-flow parameters require optimization to minimize mass transport limitation on analyte-probe interaction. Single-chain variable fragments used as analyte probes are not clinically relevant.	^14^
	dynamic range: 5–200 pg/mL		colorimetric end-point also does not offer kinetic characterization of the proteins.	
microfluidic biosensor with SERS encoding	LOD: 100 fg/mL	electrohydrodynamic nanomixing provides control over sample injection and reduces nonspecific binding.	multiple steps are required, including functionalization of a detection probe with Raman reporters.	^36^
antibody-based SPR refractometry	LOD: 5 ng/mL	the direct binding, label-free modality simplifies bioassays. The optimized regeneration protocols also enable chip reuse. Real-time binding data enables kinetic profiling, for functional characterization.	the biosensor sensitivity is low and constrained by the analyte molecular weight. Further optimizations are required to improve performance in clinical samples.	current paper
	dynamic range: 8.7–376 μg/mL			

We computed the dynamic range of the biosensor as the concentration range from the lower limit of quantification (LOQ) to the higher limit of quantification (LOQ), calculated by subtracting (*c × σ*_0_) from the upper y-asymptote of the calibration curve. From these calculations, we obtained a dynamic range of 8.74–376.45 μg/mL (∼0.33 nM–14.15 μM). Within this range, we assessed linearity at concentrations ranging from 10 to 160 ng/mL. Fitting a curve of calculated concentration versus concentration with a linear function demonstrated good linearity within this range, deriving an adjusted R-squared value of 0.995 and a slope of 0.84 with a 0.43 ng/mL offset. Additionally, the biosensor recorded acceptable accuracy/precision values (83–92%), except at 10 ng/mL (55%), indicating the reproducibility of sPD1 quantification from 20 to 160 ng/mL.^20^

### 2.5 Sensor Specificity and Selectivity

It is expected that the prepared sensor surface will exhibit minimal nonspecific binding due to the specificity of the mAb for sPD1 biomarker and the optimized surface preparation procedure. To ascertain this, we compared the plasmonic responses obtained from serial injections of sPD1 along with other cancer biomarkers commonly detected in serum: VEGF, TNF-α, and HER2, as well as BSA (Figure 6A,6B). It is also necessary to assess how the biosensor performs in the presence of serum components that would be in play with biological samples. We evaluated the accuracy of quantification of 20 ng/mL sPD1 in 1 and 10% PBS-diluted human serum. We also conducted single-cycle kinetics assays in human serum by serially diluting an sPD1-spiked 1% serum solution to final concentrations of 60, 15, and 3.75 nM. The sensorgrams obtained were double referenced against the reference flow channel and blank serum injections and fitted with the 1:1 Langmuir model. Findings of the selectivity studies are also presented in Figure 6.

**Figure 6:**
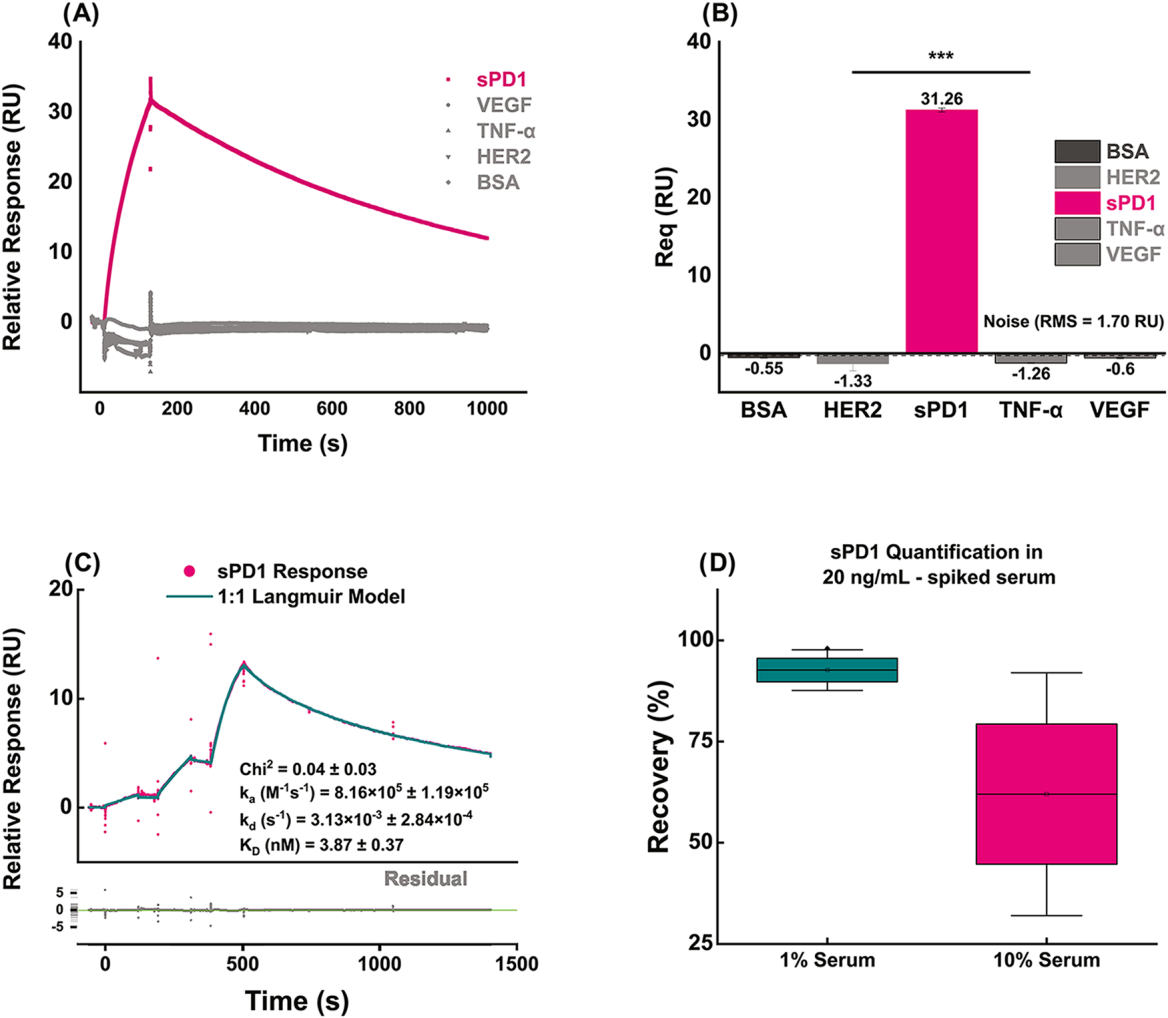
Specificity of the OPD-functionalized N-chip for sPD1 over other cancer biomarkers and *in-serum* selectivity assay findings. (A) Real-time sensorgrams of double-referenced SPR responses (reference channel and blank subtraction) for injections of sPD1 (30 nM, magenta), VEGF (60 nM, gray), TNF-α (60 nM, gray), HER2 (60 nM, gray), and BSA (0.1 mg/mL, dark gray). Each injection: 120 s contact at 30 μL/min, followed by 900 s dissociation and regeneration with 10 mM glycine–HCl (pH 3) + 2.5 M NaCl. Only sPD1 elicits a strong, sustained binding signal; all nontarget proteins yield negligible responses. (B) Bar plot of equilibrium responses (*R*_eq_; measured 10 s after injection end, averaged over *n* = 3 replicates ± SD). sPD1 produces a mean *R*_eq_ of 31.3 RU, whereas VEGF, TNF-α, HER2, and BSA show responses near zero (−1.3 to – 0.6 RU). Noise level (root-mean-square of nonspecific responses) is 1.70 RU (shaded gray band). Signal-to-noise ratio (SNR): 31.3/1.7 = 18.4. Asterisks (***) indicate statistical significance of sPD1 versus all other analytes (one-way ANOVA with Tukey’s post hoc, *p* < 0.001). (C) Averaged single-cycle kinetics sensorgram, showing sPD1 response (pink circles), following sequential injections of serially diluted sPD1-spiked serum solutions (60, 15, and 3.75 nM). Double-referenced sensorgrams exhibit robust, concentration-dependent binding, with distinct and apparent association and dissociation phases. Global 1:1 Langmuir fit (cyan line) yields *k*_a_ = 8.16 × 10^5^ ± 1.19 × 10^4^ M^–1^ s^–1^, *k*_d_ = 3.13 × 10^–3^ ± 2.84 × 10^–4^ s^–1^, *K*_D_ = 3.87 ± 0.37 nM, and *R*_max_ = 14.53 ± 0.04 RU. Goodness-of-fit statistics (χ^2^ = 0.04) and residuals (lower panel) confirm fit quality. (D) Box plots (Mean, SD, and SE) of % recovery, obtained from triplicate injections of 20 ng/mL sPD1-spiked diluted serum (Cyan box −1% Serum in PBS 1×; Pink box −10% Serum in PBS 1X). Recovery rates ranged from 88 to 98% in 1% serum, while 10% serum exhibited significant nonspecific binding, leading to overestimation of the sPD1 concentration, with recovery rates 32–92%.

The kinetic constants for sPD1–OPD binding obtained in serum within the selectivity assay remain within the range obtained in HBS-EP buffer (*k*_a_ ∼ 10^5^; *k*_d_ ∼ 10^–3^; *K*_D_ ∼ 1 nM) and other buffers reported in the literature.^26–28^ Being the first demonstration of in-serum kinetic constants calculation for the PD1-OPD interaction, this study provides evidence of the refractometric biosensor’s bimodality, incorporating functional binding analysis with conventional analyte quantification in biological samples. Recovery values are calculated in absolute terms of deviation from nominal concentration (20 ng/mL) from triplicate assays, using the equation $\left[\%\;\mathrm{re}\,\mathrm{covery}=100-(\frac{\left|20-x\right|}{20}\times 100)\right]$ , where *x* denotes the calculated concentration. The borderline high recovery values obtained in 10% serum indicate significant matrix effects, unresolved with a blocking agent (BSA), which probably arises primarily from noise-inducing serum interactions that perturb electronic activities at the atomic level and refractive indices in the macroscale during sample injection, beyond the simple RI contribution of the sPD1-OPD binding event. Substantial signal enhancement, through advanced nanoplasmonic substrate designs, and/or refractometric reporter tags in sandwich assay formats, could be pursued in subsequent studies to resolve these artifacts with better signal-to-noise performance.^20,21,37^

## 3 Conclusion

Reports have alluded to sPD1 exerting comprehensive T-cell function recovery and boosting antitumor immunity through multiple binding interactions in the immune checkpoint axis, demonstrating therapeutic effects that even supersede anti-PD1 antibodies.^38^ As a biomarker, serum sPD1 has shown promise in predicting disease progression, treatment outcomes, and the risk of developing malignant tumors, making it a hot topic in cancer biomarker research. Therefore, sPD1 quantification from liquid biopsies has an immense clinical potential. However, it is apparent that functional characterization is necessary to better contextualize and interpret sPD1-related clinical findings on a case-by-case basis.

In this study, we optimize an antibody-functionalization approach for a plasmonic chip, developing an SPR-based refractometric biosensor that, for the first time, unites label-free quantification and real-time kinetic profiling of soluble PD1 through direct binding to immobilized nivolumab. This was achieved through rational assay configurations and the reported data processing workflows, leveraging the immobilized antibody, both as a biosensing recognition element and as an analytical conjugate molecule in clinical contexts. This study presents a platform with the potential for continuous immunotherapy monitoring through simultaneous clinical quantification of immune checkpoint biomarkers and functional analyses of their antibody-binding kinetic profiles. The dual-mode SPR sensor lays a foundation for both mechanistic studies of immune checkpoint dynamics and the development of real-time monitoring tools for precision immunotherapy.

We optimized surface chemistries and regeneration strategies, developing an antibody-functionalized surface (the N-chip) which delivered dual-mode functionality, achieving a limit of detection of 5 ng/mL and a dynamic range spanning nearly five orders of magnitude, all while yielding kinetic constants in agreement with literature values in both lab-prepared buffer and dilute human serum. Despite its strengths, the sensor’s sensitivity ceiling currently precludes reliable detection of the subng/mL sPD1 levels reported in early-stage patients, and nonspecific adsorption at higher serum concentrations may compromise quantification accuracy. To overcome these limitations, future work will explore signal-amplification strategies such as localized plasmonic biosensing with nanostructured substrates or sandwich-format assays coupled with improved surface blocking to mitigate matrix effects. Extending the platform to multiplexed detection of additional immune checkpoint proteins and integrating point-of-care microfluidics could further enhance its translational impact.

The sensitivity of antibodies to ambient conditions also imposes a storage constraint on the mAb-functionalized SPR chip. Due to the reported sensitivity of proteins to pH and temperature,^16^ the OPD-functionalized chips were stored in HBS-EP 1× solutions at 4 °C. Theoretically, prolonged exposure to significantly different storage conditions is expected to induce irreversible structural damage to the surface-immobilized antibody, rendering the chip inactive. No such degradation or reduction in activity was observed with the designed chip stored under the laboratory conditions specified over 16 weeks. However, future research is still required to comprehensively profile the long-term stability of the biosensor at storage conditions more representative of the real application field.

## 4 Experimental Section

### 4.1 Materials, Reagents, and Equipment

Real-time SPR analysis was conducted using a Biacore T200 instrument (Cytiva, Sweden). The instrument was docked, as required, with either the CM5 sensor chip, a Kretschmann gold film coated with a carboxymethylated dextran matrix extending about 100 nm under physiological conditions, or the SA sensor chip, with streptavidin precoated on the carboxymethylated dextran matrix (Cytiva, Massachusetts). Prebiotinylated FcγRI (Biotinylated Human CD64 Protein, His, Avitag) was commercially sourced from ACRO Biosystems AG (Basel, Switzerland).

A stock HEPES-buffered saline with EDTA and surfactant p20 (HBS-EP 10X pH 7.4) solution was prepared by dissolution of HEPES (23.8 g), NaCl (87.6 g), EDTA (8.76 g), and surfactant p20 (500 μL) in 800 mL of distilled, 0.22 μm nitrocellulose membrane (Nitrocellulose [NC], GVS) filtered, and autoclaved water, followed by overnight stirring at 60 °C with a magnetic stirrer (Cole-Parmer, IL). The pH of the buffer was adjusted to 7.4 by dropwise addition of a 10 M NaOH solution, after which the buffer volume was increased to 1 L in a 1000 mL graduated cylinder. The stock buffer was stored in a corked jar at room temperature, and 10-fold dilutions were made to prepare the required volumes of HBS-EP 1× buffer (pH 7.4), which was used as the running buffer for all experiments except in-serum kinetics and selectivity studies. Stock phosphate-buffered saline (PBS 10× pH 7.4) containing 0.1 M phosphate buffer, 27 mM KCl, and 1.37 M NaCl was procured from GE Healthcare Bio-Sciences AB (Uppsala, Sweden). A 10-fold dilution of the PBS stock was prepared in distilled, 0.22 μm nitrocellulose membrane-filtered, and autoclaved water to create PBS 1× pH 7.4 for the selectivity and in-serum kinetics assays.

Nivolumab sterile intravenous infusion (Opdivo, Bristol Myers Squibb) was obtained commercially from a retail pharmacy outlet (Istanbul, Türkiye). A 1 μM working solution was prepared in HBS-EP buffer 1× (pH 7.4) and stored at 4 to 8 °C, with onward dilutions in HBS-EP 1× buffer (pH 7.4) to desired concentrations for the various experiments. Similarly, human sPD1 protein (ECD, HisTag) was purchased from Sino Biological Europe GmbH (Eschborn, Germany) and stored at −20 °C. Human serum was prepared by clotting, centrifugation, and supernatant extraction of blood obtained from a healthy adult volunteer. Prepared serum was stored at −20 °C until use.

### 4.2 Sensor Surface Preparation

The SPR biosensor setup is designed to leverage the high affinity and specificity of nivolumab (OPD) for the PD1 ectodomain. It is consequently adopted as a bioconjugate probe molecule for detecting the sPD1 analyte. Accordingly, two approaches were assessed. A first approach explored capturing the mAb on an FcγRI-immobilized surface, leveraging interactions between the mAb Fc and FcγRI to achieve site-oriented capture (the *F-chip*). A second approach involves directly immobilizing the OPD molecules on the SPR chip by covalent bonds constituting the *N-chip*.

#### 4.2.1 F-Chip Preparation

Biotinylated FcγRI was immobilized on the sensor chip SA following the streptavidin–biotin capture method.^20^ The SA chip was inserted and docked on the Biacore T200 instrument (Cytiva, Sweden), after which the signal was normalized using a 70% glycerol solution,^25^ and the sensor surface was primed with the running buffer. Subsequently, the sensor surface was conditioned with three 60 s injections of a 1 M NaCl and 50 mM NaOH solution at 10 μL·min^–1^, ridding the surface of contaminants, stabilizing the signal baseline, and preparing the surface for protein binding. Biotinylated FcγRI ectodomain was injected into the active flow channel (Fc2) in pulses, with an immobilization target of 150 Response Units (RU). Nonspecifically bound materials were removed, and the immobilized surface was stabilized by washing with a 50% solution of isopropanol in 50 mM NaOH/1 M NaCl.^25^ A blank immobilization run was done to prepare a reference channel (Fc1), in which all of the steps conducted for Fc2 were repeated, but without the biotinylated FcγRI injection step.

Subsequently, OPD was injected on both channels at concentrations of 90, 30, and 10 nM for 120 s contact time and 900 s dissociation time. The sensor surface was regenerated on this occasion using 10 mM glycine–HCl buffer (pH 3.0), following a literature-reported regeneration method.^20^ The relative responses obtained were double referenced against both the reference flow channel (Fc2–1) and blank injection cycles (injection of blank running buffer as a sample). Using the Biacore Evaluation software (3.0 Biacore T200, Shrewsbury, MA), kinetic parameters of the binding of the OPD-FcγRI were calculated with the 1:1 Langmuir binding model. Steady-state affinity (*K*_D_) was also determined using the software’s steady-state affinity algorithm.

#### 4.2.2 N-Chip Preparation

The N-chip was prepared by direct covalent immobilization of the OPD on a sensor chip (CM5, Cytiva, Sweden), which contains carboxymethyl groups in its dextran matrix. Amine coupling chemistry was adopted to prepare the N-chip, in which the sensor surface was activated with an equal parts mixture of ethyl-(dimethylaminopropyl)-carbodiimide (EDC) and *N*-hydroxysuccinimide (NHS), followed with an injection of a 50 nM solution of OPD in 10 mM acetate buffer (pH 5.0), in pulses until the specified immobilization target was reached. Residual activated carboxyl groups in the dextran matrix were inactivated with an injection of ethanolamine hydrochloride.

### 4.3 Sensor Surface Regeneration

Mass changes at the surface of a typical SPR refractometric sensor result in refractive index alterations of the reaction medium, generating resonance wavelength shifts that are quantified as a measure of biomolecular interactions between an injected analyte and an immobilized ligand. Crucially, the reusability of such a setup requires restoration of the sensor surface in preparation for a new cycle. Quantitatively, the ideal sensor surface would maintain an inalterable baseline response without considerable damage to the binding capacity of the surface-immobilized ligand, having been subjected to a regeneration program.

Typically, high/low pH solutions, high ionic strength solutions, and low concentration detergent (sodium dodecyl sulfate) are some of the solutions injected over the sensor surface for regeneration.^25^ However, a condition that optimally regenerates the sensor surface depends on the ligand–analyte pair, immobilization chemistry, and assay conditions, among other factors specific to the peculiar setup. It is therefore necessary to experimentally determine the optimum regeneration conditions for the biosensor setup. We optimized the regeneration conditions for both setups under study through a series of successive regeneration studies encompassing both scouting and verification.

#### 4.3.1 F-Chip Regeneration

Six distinct regeneration conditions were assessed in the scouting phase to identify potentially suitable conditions for regenerating the F-chip surface. The conditions scouted include 10 mM glycine–HCl buffer (pH 3.0), 10 mM acetate buffers (pH 4.5, 5.0, and 5.5), 5 M sodium chloride (NaCl), and 100% ethylene glycol. Following capture of the OPD (90 nM) over the FcγRI surface at a 30 μL·min^–1^ flow rate injection for 120 s, the regeneration solution was injected for 30 s, allowing a 60 s stabilization period. This sequence was repeated five times for each regeneration condition. Subsequently, baseline and equilibrium binding responses (recorded as the absolute response 10 s before the start of OPD injection and relative/baseline-subtracted response 10 s after OPD injection completion, respectively, over 5 s windows) were recorded and compared among the regeneration conditions to select the conditions to progress to regeneration verification. Additionally, an extra regeneration condition was established to simulate spontaneous regeneration, wherein a blank running buffer was injected over a 300 s contact time, leveraging literature reports of the high dissociation rate constant (*k*_d_) of the interaction between FcγRI and OPD-Fc region, being an IgG4-type mAb.^23^

Based on the results of the regeneration scouting exercise, two regeneration conditions were advanced to the regeneration verification stage, including 10 mM glycine–HCl buffer and 100% ethylene glycol. In this study, each condition was applied to regenerate the sensor surface for 30 cycles, comprising 15 blank injection cycles and 15 OPD injection cycles. During these cycles, a 90 nM OPD solution was injected for 120 s, followed by a 900 s dissociation period. Each regeneration condition was injected for 30 s at 30 μL·min^–1^, allowing a 60 s stabilization period after regeneration.

#### 4.3.2 N-Chip Regeneration Studies

The N-chip regeneration condition was optimized similarly to that of the F-chip. Based on existing models of the chemistry underlying the interaction of the PD1 ectodomain with OPD reported in the literature,^28^ three regeneration conditions were scouted, including a sequential injection of 10 mM glycine–HCl buffer (pH 3) followed by 2.5 M NaCl solution (glycine + NaCl); a similar sequential injection of 10 mM acetate buffer (pH 5), followed by 2.5 M NaCl solution (acetate + NaCl); third, a single injection of 5 M NaCl solution. Each condition was applied to regenerate the sensor surface for five consecutive cycles following the injection of a 60 nM sPD1 solution over the sensor surface. This was achieved by injecting 30 μL·min^–1^ for 30 s, followed by a 60 s stabilization period. Based on the results of the regeneration scouting, verification studies were conducted for two conditions; glycine + NaCl and 5 M NaCl, where each condition was applied to regenerate the sensor surface in 30 consecutive cycles, 15 of which included injection of a 60 nM OPD solution, and the other 15 included blank running buffer injections for 120 s contact and 900 s dissociation time.

### 4.4 sPD1 Detection and Quantification

#### 4.4.1 sPD1 Detection and Kinetics Assay

A single-cycle kinetic assay was conducted to assess the antibody-binding behavior of the sPD1 analyte. On the F-chip, OPD was first captured by injecting a 90 nM solution over the FcγRI-immobilized surface for 120 s at a 30 μL·min^–1^ flow rate. Subsequently, sPD1 was injected at concentrations of 90, 18, and 3.6 nM for 120 s at 30 μL·min^–1^, allowing spontaneous sensor surface regeneration over a dissociation period of 1800 s. In contrast, sPD1 was injected at concentrations of 60, 15, and 3.75 nM over the OPD-immobilized N-chip surface for a 120 s contact time at 30 μL·min^–1^, allowing a 900 s dissociation time. The sensor surface was regenerated with the glycine + NaCl regeneration condition developed in v 4.3.2.

The assay was conducted in triplicate for each sensor chip, and the kinetic constants characterizing the OPD-sPD1 binding interaction were obtained using Biacore Evaluation software (version 3.0, Biacore T200, Shrewsbury, MA) by fitting the obtained sensorgrams with a 1:1 Langmuir binding model.

#### 4.4.2 sPD1 Quantification/Sensor Sensitivity

Quantification analysis was conducted by obtaining a calibration curve to model the sPD1 concentration response of the sensor. For this purpose, six serial concentrations of sPD1:1500, 150, 15, 1.5, 0.15, and 0.015 ng/mL were injected over both active and reference channels of the sensor chip in separate cycles. A blank running buffer, corresponding to 0 ng/mL sPD1 concentration, was also injected. Each solution was injected in separate cycles over 180 s at a 10 μL·min^–1^ flow rate, and the response at equilibrium (*R*_eq_) obtained with each concentration was recorded as the response received, 10 s after the completion of sample injection, over a 5 s window. Each injection cycle was performed in triplicate, and a plot of average *R*_eq_ against concentration was fitted with a four-parameter logistic function to obtain the calibration curve. Additionally, samples of sPD1 at concentrations of 10, 20, 40, 80, and 160 ng/mL were injected under the same conditions to assess the linearity of the sensor within the established dynamic range, based on the optimized experimental conditions.

### 4.5 Sensor Specificity and Selectivity

The specificity of the OPD-functionalized sensor for sPD1 is an important determinant of its diagnostic performance. To ascertain the absence of significant nonspecific or promiscuous binding of diverse proteins by the sensor surface, its specificity for sPD1 was assessed by injecting the sPD1 protein alongside other serum cancer biomarkers including vascular endothelial growth factor (VEGF), tumor necrosis factor (TNF-α), human epidermal growth factor receptor 2 (HER2), at 60 nM, and a 0.1 mg/mL bovine serum albumin (BSA) solution, in different respective cycles for 120 s, allowing a 900 s dissociation time. The sensor surface was regenerated with glycine and NaCl after each cycle. Double-referenced *R*_eq_ obtained with each protein was averaged over three replicates. The specificity of the sensor was assessed by the statistical significance of the response obtained with sPD1 compared to other proteins. Considering the plasmonic response obtained with the other proteins as electrical noise, a signal-to-noise ratio (SNR) was computed for the sensor to quantify its specificity for sPD1 and its detection reliability.

To further evaluate the performance of the biosensor in sPD1 detection within biological samples, a selectivity experiment was conducted. Prepared human serum was spiked with sPD1 (20 ng/mL) and injected over the OPD-functionalized chip at 10 μL·min^–1^ for 180 s. The sensor was regenerated with the glycine + NaCl regeneration procedure developed earlier, and responses obtained from the spiked serum injection were double-referenced against responses in the reference flow cell and blank serum injections, using blank human serum as the running buffer. The selectivity experiment was conducted in 1 and 10% human serum diluted with PBS 1× pH 7.4 buffer.^39,40^ Additionally, a proof of concept for the multifunctionality of the biosensor in a biological matrix was conducted through a single-cycle kinetics assay of 1% dilute human serum spiked with sPD1 (60 nM). The spiked serum was serially diluted 4× in two steps to attain final concentrations of 15 and 3.75 nM, which were injected following the same procedure adopted in 4.4.1, with blank 1% human serum as the running buffer. Before each experiment, the prepared dilute serum was injected over the sensor chip at a 45 μL/min flow rate for at least 24 h to equilibrate the SPR detector and ensure complete evacuation of residual HBS-EP buffer from previous experiments from the flow system.

## Abbreviations

EDC: ethyl-(dimethylaminopropyl)-carbodiimide
ELISA: enzyme-linked immunosorbent assay
FcγR: Fc-γ receptor
IgG: immunoglobulin
LOD: limit of detection
LOQ: limit of quantification
mAb: monoclonal antibody
NHS: *N*-hydroxysuccinimide
OPD: opdivo/nivolumab
PD1: programmed cell death protein 1
PD-L1: programmed cell death ligand 1
PD-L2: programmed cell death ligand 2
SERS: surface-enhanced Raman spectroscopy
sPD1: soluble PD1
SPR: surface plasmon resonance
TNF-α: tumor necrosis factor-α
VEGF: vascular endothelial growth factor
